# Engineering a Novel Antibody-Peptide Bispecific Fusion Protein Against MERS-CoV

**DOI:** 10.3390/antib8040053

**Published:** 2019-11-04

**Authors:** Lili Wang, Jiyan Xu, Yu Kong, Ruiying Liang, Wei Li, Jinyao Li, Jun Lu, Dimiter S. Dimitrov, Fei Yu, Yanling Wu, Tianlei Ying

**Affiliations:** 1 MOE/NHC/CAMS Key Laboratory of Medical Molecular Virology, School of Basic Medical Sciences, Shanghai Medical College, Fudan University, Shanghai 200032, China; liliwang@fudan.edu.cn (L.W.); 13301050116@fudan.edu.cn (J.X.); kongyu@fudan.edu.cn (Y.K.); tlying@fudan.edu.cn (T.Y.); 2 Research Center of Chinese Jujube, Hebei Agricultural University, Baoding 071001, China; 3 College of Life and Science, Hebei Agricultural University, Baoding 071001, China; ruiyingliang@outlook.com; 4 Center for Antibody Therapeutics, University of Pittsburgh Medical School, Pittsburgh, PA 15261, USA; LIWEI171@pitt.edu (W.L.); dsd116@pitt.edu (D.S.D.); 5 Xinjiang Key Laboratory of Biological Resources and Genetic Engineering, College of Life Science and Technology, Xinjiang University, Urumqi 830046, China; ljyxju@xju.edu.cn; 6 School of Science, and School of Interprofessional Health Studies, Faculty of Health & Environmental Sciences, Auckland University of Technology, Auckland 1142, New Zealand; jun.lu@aut.ac.nz

**Keywords:** MERS-CoV, mAbs, polypeptides, bispecific, immunotherapeutics

## Abstract

In recent years, tremendous efforts have been made in the engineering of bispecific or multi-specific antibody-based therapeutics by combining two or more functional antigen-recognizing elements into a single construct. However, to the best of our knowledge there has been no reported cases of effective antiviral antibody-peptide bispecific fusion proteins. We previously developed potent fully human monoclonal antibodies and inhibitory peptides against Middle East Respiratory Syndrome Coronavirus (MERS-CoV), a novel coronavirus that causes severe acute respiratory illness with high mortality. Here, we describe the generation of antibody-peptide bispecific fusion proteins, each of which contains an anti-MERS-CoV single-chain antibody m336 (or normal human IgG1 CH3 domain as a control) linked with, or without, a MERS-CoV fusion inhibitory peptide HR2P. We found that one of these fusion proteins, designated as m336 diabody-pep, exhibited more potent inhibitory activity than the antibody or the peptide alone against pseudotyped MERS-CoV infection and MERS-CoV S protein-mediated cell-cell fusion, suggesting its potential to be developed as an effective bispecific immunotherapeutic for clinical use.

## 1. Introduction

The antibody-based therapeutic modalities have shown clinical success in the treatment of many diseases [1,2,3,4]. In recent years, tremendous efforts have been made in the engineering of bispecific or multi-specific antibodies by combining two or more functional antigen-recognizing elements into a single construct [5,6]. Such novel antibodies, or antibody-based fusion proteins, could be particularly beneficial for the treatment of viral infections, which typically require potent and multi-functional therapeutics to prevent the frequent incidence of viral escape mutants [7]. For instance, we previously engineered a bispecific and multivalent anti-HIV-1 fusion protein, by incorporating the HIV-1 neutralizing antibody and the engineered single-domain CD4 into a single antibody-like molecule, and found that it was able to neutralize all tested HIV-1 isolates, mediate potent antibody-dependent cellular cytotoxicity (ADCC) against HIV-1-infected cells, and effectively suppress HIV-1 or SHIV infection in humanized mice and chronically infected macaques [8,9,10]. Notably, in addition to antibody-based therapeutics, the polypeptides-based fusion inhibitors represent another type of effective antivirals, that could inhibit the entry of viruses by inhibiting virus-mediated cell-cell fusion [11]. However, due to the vast differences in activity, bioavailability, and biophysical properties between polypeptides and monoclonal antibodies, there has been no reported case of antibody-peptide bispecific fusion protein that is able to effectively neutralize and inhibit cell-cell fusion mediated by viruses.

The Middle East respiratory syndrome coronavirus (MERS-CoV) is a novel coronavirus first isolated in September 2012 from a patient in Saudi Arabia [12,13]. It causes SARS-like symptoms, including fever, cough, shortness of breath, and can lead to respiratory or renal failure [14,15]. Bats are natural reservoirs of MERS-CoV, but it is predominantly transmitted via dromedary camels and humans [16,17,18,19,20,21,22,23]. At the end of May 2019, 27 countries have reported 2442 laboratory-confirmed cases of MERS-CoV infections with at least 842 related deaths since September 2012 (http://www.who.int/emergencies/mers-cov/en/). The effective therapeutics and vaccines are urgently needed, considering the possibility of evolution and pandemic potential of MERS-CoV [24,25,26,27].

Like SARS-CoV, MERS-CoV is an enveloped virus using its spike (S) protein to enter target cells. The S protein can be cleaved into two subunits, S1 and S2 whereby the S1 subunit binds to the cellular receptor DPP4 and S2 subunit mediates membrane fusion [28,29,30,31,32]. Therefore, both S1 and S2 subunits could be targets for the development of prophylactic and therapeutic agents against MERS-CoV infection [33]. In previous studies, by screening a large phage-displayed antibody Fab library, we have identified a panel of human neutralizing monoclonal antibodies (mAbs) targeting the receptor binding domain (RBD) of the MERS-CoV S protein S1 subunit. Among these antibodies, the mAbs m336 showed the most potent virus neutralization activity at low nanomolar concentrations [34,35,36,37]. Further structural study indicated that the binding epitope of m336 on MERS-CoV almost completely overlapped with the viral receptor-binding site, revealing the mechanism for its high neutralizing potency [38]. Meanwhile, fusion inhibitory peptides derived from heptad repeat 2 domain (HR2) of MERS-CoV S protein S1 subunit can inhibit the formation of six helix bundles (6-HB), which are required for fusion of the virus with its target cell [39,40]. Two of the peptides, P1 and HR2P, were reported to interact with heptad repeat 1 domain (HR1) of S protein S2 subunit, to form a 6-HB complex and block viral fusion and replication [40]. It was recently found that the combination of m336 antibody and HR2P peptide exhibited potent synergism in inhibiting MERS-CoV S protein-mediated cell-cell fusion and pseudovirus infections [41]. However, the peptide was mixed with antibody at a molar concentration ratio of 10,000:1, and no synergic effect was observed when mixing the peptide with antibody at a ratio of 1:1, probably due to the significant difference between the peptide and antibody in their bioactivities and biophysical properties.

## 2. Materials and Methods

### 2.1. Gene Construction

Four constructs, m336 scFv, m336 scFv-pep, m336 diabody-pep, and CH3-pep were generated. The m336 scFv was generated by linking the m336 variable region heavy and variable region light chains with a 15 amino acids (G_4_S)_3_ linker. Fusion protein m336 scFv-pep was prepared by linking m336 scFv with MERS-CoV-derived HR2P peptide (LTQINTTLLDLTYEMLSLQQVVKALNESYIDLKEL) using another (G_4_S)_3_ linker as the spacer. Fusion protein m336 diabody-pep was generated by replacing the (G_4_S)_3_ linker between VH and VL of m336 scFv-pep with a 5 amino acids GGGGS linker, so that two VHs and VLs can pair together to form the dimeric diabody. The CH3-pep was also generated by fusing HR2P peptide with IgG1 CH3 domain. Coding fragments were synthesized by Genewiz Biotech Co., and inserted between two SfiI restriction sites in the pComb3x vector with Flag-tag (DYKDDDDK) and His_6_-tag on C-termini. The sequences of the constructs were verified by direct DNA sequencing.

### 2.2. Protein Expression and Purification

All four constructs were expressed as soluble proteins in *E. coli* and purified on Ni-NTA column. Briefly, the expression vectors were transformed into *E. coli* strain HB2151 competent cells. A single fresh colony was inoculated into 3 mL of 2YT medium containing 100 μg/mL ampicillin and incubated at 37 °C, 250 rpm overnight. The incubated culture was transferred to 200 mL of fresh 2YT medium with 100 μg/mL ampicillin for large-scale protein production and 4–6 h growth at 37 °C. Then, 1 mM isopropyl-β-d-thiogalactoside (IPTG) was applied to induce protein overexpression, and the cells were grown overnight at 30 °C before harvesting by centrifugation. Protein fragments were harvested from the bacterial cell pellet and the precipitate was re-suspended with 50 mL PBS with 0.5 M NaCl. Then, 0.2 mL 1 M polymyxin B was added to lyse bacteria and the sample was rotated 30 min at room temperature, 250 rpm. The cultures were centrifuged at 12,000 rpm for 15 min at 4 °C. Supernatant was collected and loaded onto a Ni-NTA Superflow (Qiagen, Redwood City, CA, USA). After the impurities were removed, target proteins were eluted with elution buffer (250 mM imidazole in PBS). The proteins were resolved with SDS-PAGE and the purity was estimated as >90%. The protein purity was further confirmed by size exclusion chromatography using an FPLC AKTA system (GE Healthcare, Chicago, IL, USA) with a Superdex 200 10/300 GL column (GE Healthcare). The protein concentration was measured spectrophotometrically (NanoVue, GE Healthcare).

### 2.3. MERS-CoV S Protein Binding

The experiments were performed using the ProteOn XRP36 system (Bio-Rad, Hercules, CA, USA) to measure the binding kinetics of m336 scFv, m336 scFv-pep, m336 diabody-pep, and CH3-pep to MERS-CoV S protein (amino acid 18–725). The S protein was immobilized on a ProteOn GLM biosensor chip using standard amine coupling chemistry (300 nM in 10 mM sodium acetate buffer, pH 5.0), and ~3000 resonance units were immobilized. The surface of the sensor chip was activated with 200 mM 1-ethyl-3-dimethyl aminopropylcarbodiimide hydrochloride and 50 mM N-hydroxysulfosuccinimide. m336 scFv, m336 scFv-pep, m336 diabody-pep, and CH3-pep were prepared in PBS, pH 7.4, containing 0.005% Tween-20 (PBS-T) and injected (50 μL/min for 120 s, 1:3 dilution from 200 nM). The dissociation phase was followed for 600 s and chip surfaces were regenerated by injecting 10 mM glycine HCl, pH2.0, 100 μL/min for 18 s. Data were analyzed using ProteOn Manager 3.1 software and fitted to the 1:1 interaction model [42].

### 2.4. MERS-CoV Neutralization Assay

MERS-CoV neutralization assay was performed as previously described [43,44]. Briefly, 293T cells in 10 cm^2^ dishes were transiently co-transfected with a pcDNA3.1-MERS-CoV-S plasmid and a PNL4-3.luc.RE plasmid encoding an Env-defective luciferase-expressing HIV-1genome. After 48 h post-transfection, the produced pseudovirus was harvested from the supernatant, and filtered through 0.45 μm sterilized membrane. The MERS-CoV pseudovirus was incubated with four inhibitors at 37 °C for 30 min, and then pseudovirus and inhibitors were added to DPP4-expressing Huh-7 cells (10^4^/well) preplated in 96 well tissue culture plates for 6 h. After 12 h, fresh medium was added to the plates and incubated for another 48 h. Cells were lysed with lysis reagent (Promega, Madison, WI, USA) and lysates were transferred into 96-well Costar flat-bottom luminometer plates (Corning, Corning, NY, USA). Luciferase substrate was added and the readings were recorded with an Ultra 384 Microplate Reader (Tecan, Männedorf, Switzerland).

### 2.5. Cell-Cell Fusion Assay

To further compare the inhibitory effects of fusion proteins, the cell-cell fusion assay was performed as MERS-CoV S protein, which was expressed on the cell surface, can mediate cell fusion with neighboring cells [43]. First, 293T cells were transiently transfected with pAA-IRES-MERS-EGFP (293T/MERS/EGFP, effector cells) encoding the MERS-CoV proteins or pAA-IRES-EGFP (293T/EGFP) as a negative control. Cells were cultured in DMEM containing 10% FBS at 37 °C for 48 h. Preplated Huh-7 cells (10^4^, target cells), which express the DPP4 MERS-CoV receptor, were cultured in 96 well plates at 37 °C for 5 h. After addition of 293T/MERS/EGFP or 293T/EGFP cells with 2-fold diluted m336 scFv, m336 scFv-pep, m336 diabody-pep, or CH3-pep from 2.5 μg/mL, the mixture and Huh-7 cells (target cells) were further cultures at 37 °C for 4 h, and fused or unfused cells were visualized under an inverted fluorescent microscope (Nikon Eclipse Ti-S100, Nikon, Tokyo, Japan) [45].

## 3. Results

### 3.1. Generation of Anti-MERS-CoV Fusion Proteins

**Figure 1:**
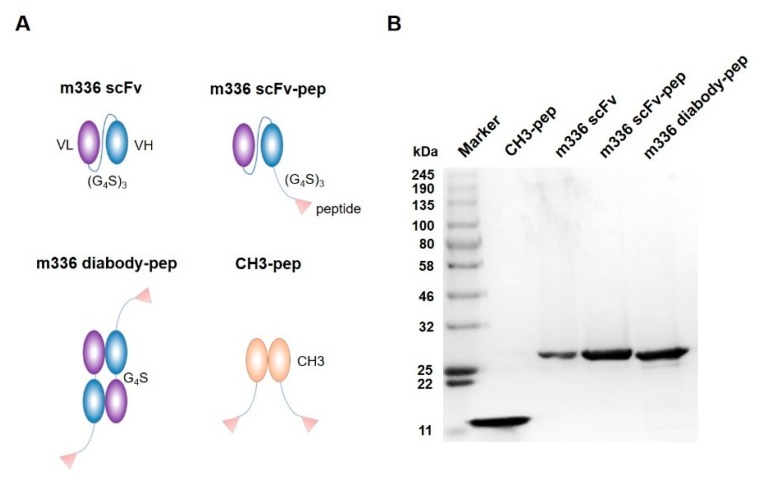
Construction and characterization of anti-MERS-CoV inhibitors. (**A**) Schematic structure of proteins m336 scFv, m336 scFv-pep, m336 diabody-pep, and CH3-pep. (**B**) SDS-PAGE of the anti-MERS-CoV inhibitors.

To generate the bispecific antibody-peptide inhibitor, the MERS-CoV S2-derived peptide HR2P was fused to m336 scFv using a flexible (G_4_S)_3_ linker. Furthermore, a dimeric format, namely m336 diabody-pep, was also generated by replacing the (G_4_S)_3_ linker between variable region heavy (VH) and variable region light (VL) of m336 scFv with a 5 amino acids GGGGS linker. The shorter linker can result in the pairing of VH and VL from two different chains and thus the formation of dimeric diabody with two VHs and VLs. A dimeric peptide-only control, namely CH3-pep, was also generated by linking HR2P peptide and IgG1 CH3 domain with the flexible (G_4_S)_3_ linker. This fusion protein possesses two peptides and comparable molecular weight compared to other constructs (Figure 1A).

The four proteins, m336 scFv, m336 scFv-pep, m336 diabody-pep, and CH3-pep were soluble expressed in *E. coli* cells with high efficiency. Purified proteins were obtained with yields of 15–30 mg/L bacterial culture. The m336 diabody-pep migrated as a monomer under the denaturing conditions of the SDS-PAGE, and thus displayed similar molecular weight (~30 kDa) with m336 scFv and m336 scFv-pep (Figure 1B). All proteins were monomeric as demonstrated by size exclusion chromatography (data not shown).

### 3.2. Interactions between Fusion Proteins and MERS-CoV S Protein

**Figure 2:**
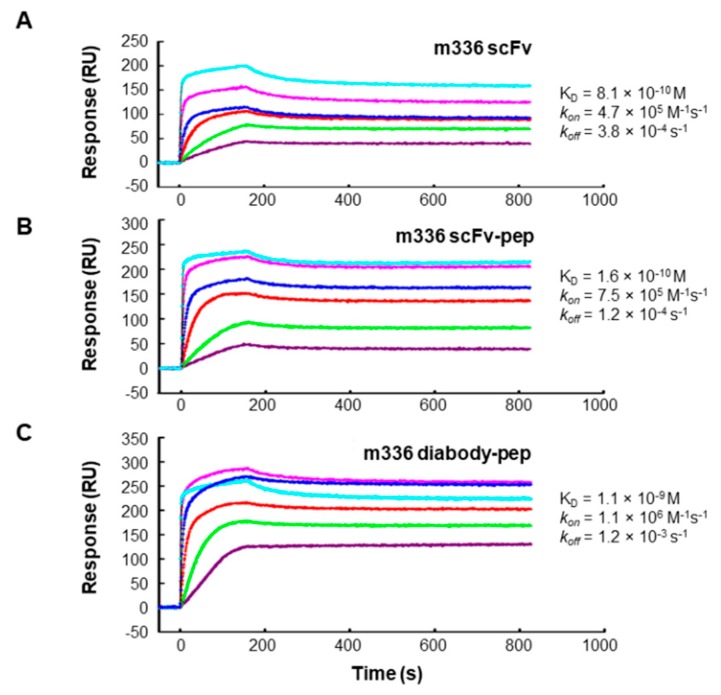
Interactions between m336 scFv (**A**), m336 scFv-pep (**B**), and m336 diabody-pep (**C**) with MERS-CoV S protein measured using the ProteOn XRP36 SPR system.

To measure the binding kinetics of the fusion proteins with MERS-CoV S protein, the surface plasmon resonance (SPR) assay was performed by using a ProteOn system SPR biosensor instrument (Figure 2). We have previously demonstrated that m336 bound specifically to MERS-CoV S protein, but not other unrelated proteins [35]. As shown in Table 1, all three m336-based fusion proteins (m336 scFv, m336 scFv-pep, and m336 diabody-pep) exhibited potent binding to S protein with very similar binding pattern. The equilibrium dissociation constant (K_D_) of m336 scFv for S protein was 0.8 nM with on-rate (*k*_on_) of 4.7 × 10^5^ M^−1^s^−1^ and off-rate (*k*_off_) of 3.8 × 10^−4^ s^−1^. The m336 diabody-pep displayed similar binding kinetics to that of m336 scFv (*k*_on_ 1.1 × 10^6^ M^−1^s^−1^, *k*_off_ 1.2 × 10^−3^ s^−1^, K_D_ 1.1 nM). The m336 scFv-pep had slightly more potent binding affinity (K_D_ 0.2 nM) with slower dissociation rate constant (*k*_off_ 1.2 × 10^−4^ s^−1^), compared to the other two fusion proteins. In contrast, the CH3-peptide displayed much lower affinity, compared to the antibody m336-based fusion proteins (K_D_ 1.1 M).

### 3.3. Neutralization of MERS-CoV Pseudovirus Infection

**Figure 3:**
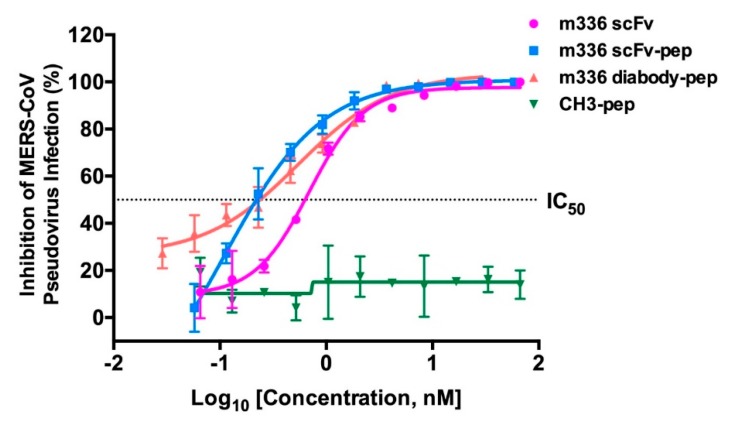
Potent neutralization of MERS-CoV by anti-MERS-CoV inhibitors. Pseudotyped virus was incubated with proteins before infection of DPP4-expressing Huh-7 cells. Luciferase activities were measured, and percent neutralization was calculated.

Next, we measured the neutralization activity of m336 scFv, m336 scFv-pep, m336 diabody-pep, and CH3-pep at graded concentrations against pseudotyped MERS-CoV infection. As shown in Figure 3, m336-scFv, m336 scFv-pep and m336 diabody-pep exhibited potent neutralization activity. Among them, m336 scFv-pep exhibited the most potent neutralization capability, with 50% inhibitory concentration (IC_50_) of 0.21 ± 0.06 nM. Although, displaying comparable IC_50_ (m336 diabody-pep, 0.25 ± 0.07 nM; m336 scFv, 0.69 ± 0.03 nM), interestingly, m336 diabody-pep inhibited the infection more potent than that of m336 scFv at low protein concentrations (<0.03 nM). In a previous study, IgG1 m336 displayed exceptional neutralization against pseudotyped MERS-CoV infection with IC_50_ of 0.03 nM [35]. The control fusion protein CH3-pep could not inhibit the infection.

### 3.4. Inhibition of MERS S Protein-Mediated Cell-Cell Fusion

**Figure 4:**
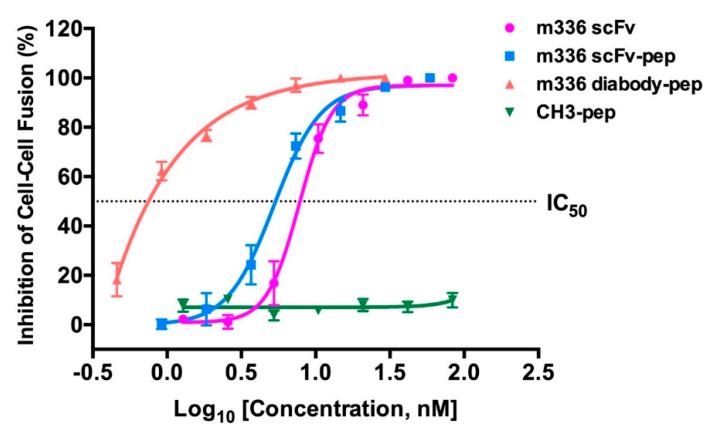
Inhibitory activity of anti-MERS-CoV inhibitors on MERS-CoV S protein-mediated cell-cell fusion. The number of 293T/MERS/EGFP cells fused or unfused with Huh-7 cells were counted, and the percentage of inhibition was calculated.

We used a well-established MERS-CoV S protein-mediated cell-cell fusion assay to determine whether the fusion proteins have the ability to inhibit MERS-CoV fusion with the target cells [40,46]. In such assay, the 293T cells that express MERS-CoV S protein and EGFP were used as the effector cells, and Huh-7 cells that express DPP4 receptor were used as the target cells. The CH3-pep showed no appreciable activity at a concentration of 83 nM, which is consistent with the previous report that the HR2P peptide did not have significant inhibitory effect at concentrations lower than 100 nM [40]. Notably, we found that m336 diabody-pep was able to block the fusion between 293T/MERS/EGFP cells and Huh-7 cells at a concentration as low as 0.5 nM (Figure 4). The calculated IC_50_ for m336 diabody-pep was 0.81 ± 0.02 nM. As shown in Figure 4, the m336 diabody-pep was significantly more potent than m336 scFv in inhibiting cell-cell fusion. The m336 scFv-pep also showed evidently more potent inhibitory activity than m336 scFv at low concentrations, with slightly lower IC_50_ (m336 scFv-pep, 5.25 ± 0.03 nM; m336 scFv, 7.76 ± 0.02 nM). These results indicate that antibody-peptide bispecific fusion proteins, especially the m336 diabody-pep, have the potential to be developed as potent MERS-CoV inhibitors that could, not only neutralize MERS-CoV infection, but also inhibit MERS-CoV S protein-mediated cell-cell fusion.

## 4. Discussion

Monoclonal antibodies represent one of the most promising immunotherapeutic agents for the treatment of cancers and infectious diseases [7,47,48,49,50,51,52]. Previously, we used RBD of MERS-CoV S protein to screen a non-immune phage-displayed Fab library and identified a highly potent human neutralizing mAb m336 and a panel of other mAbs against MERS-CoV [34,36,37]. Jiang et al. also used MERS-CoV S RBD to screen a non-immune yeast-displayed scFv library and identified two potent MERS-CoV neutralizing mAbs, MERS-4 and MERS-27 [53]. Tang et al. used a full-length S protein to screen a non-immune phage-display scFv library and identified a potent MERS-CoV neutralizing mAb 3B11 [54]. All these reported mAbs inhibit the binding of virus to cellular reporter DPP4 by recognizing the different epitopes of RBD, thus having the potential to be developed as anti-MERS-CoV therapeutics. However, further engineering is still needed to address the concerns in using such mAbs in clinics, e.g., the high production costs, etc.

In this study, we generated two bispecific anti-MERS-CoV fusion proteins, m336 scFv-pep and m336 diabody-pep. Compared with the antibody or peptide inhibitors that block only one site of the spike protein, the bispecific inhibitors showed greater potency. The m336 scFv-pep showed the highest binding affinity to MERS-CoV S protein, as well as the most potent neutralizing activity, while the m336 diabody-pep was the most potent in blocking MERS-CoV S-mediated cell-cell fusion. These results confirmed the improved potency of bispecific antibody-peptide inhibitors compared to antibody or peptide alone. The m336-scFv interferes with viral attachment to the DPP4 receptor on human cells [35], while the HR2P peptide inhibits the formation of the 6-helix-bundle fusion core and thus interrupts viral fusion with the target cell membrane [40]. It is probably that the antibody-peptide fusion proteins, which are composed of both antiviral components, linked by flexible (G_4_S)_3_ linker, can act at both steps during viral infection.

Interestingly, the m336 diabody-pep was found to potently block MERS-CoV S-mediated cell-cell fusion even at concentrations lower than 0.5 nM. This phenomenon may be attributed to the difference in steric hindrance of the fusion proteins. Compared to the m336 scFv-pep, the m336 diabody-pep has a much larger (twice) molecular weight and size, and thus, inhibits the viral fusion to target cells by competitive inhibition and steric hindrance, leading to the much greater inhibitory activity than that of m336 scFv-pep. Although, having largely improved potency in inhibiting cell-cell fusion, m336 diabody-pep did not show evidently increased neutralization activity compared to m336 scFv. Further investigations are required to explore the mechanism for the disparate performance of m336 diabody-pep in different assays. As shown in Figure 3 and Figure 4, the IC_50_ of m336 diabody-pep was 0.25 ± 0.07 nM in the neutralization assay, and 0.81 ± 0.02 nM in the cell-cell fusion assay. Importantly, m336 diabody-pep was much more potent than m336 scFv at concentrations higher than 5 nM in the cell-cell fusion assay. At these concentrations, all the fusion proteins can result in high percentage of inhibition (>70%) in the neutralization assay, and therefore, may explain the little difference among difference groups.

All the m336-based fusion proteins could be easily produced in *E. coli* in large amount and low production cost. Their sizes are larger than m336 scFv or HR2P peptides, suggesting that they would possess longer in vivo half-life. Still, their half-life would be shorter than full-size IgG due to the lack of Fc region. Therefore, their prophylactic and therapeutic efficacy against MERS-CoV should be assessed carefully using different approaches and doses in vivo. However, the RBD of MERS-CoV specifically targets on human DPP4 [29], and most small animal models are not susceptible to MERS-CoV infection [55], which pose a significant barrier to the development of anti-MERS-CoV inhibitors. Fortunately, researchers have constructed several animal models recently that simulate the morbidity and mortality of human infections, of which nonhuman primates (NHP) models and human DPP4-expressing mouse model are considered to be ideal candidates [56] and the latter is promising to be utilized in our following studies.

## 5. Conclusions

In conclusion, our study indicated that the bispecific inhibitors have increased the efficacy against MERS-CoV, compared to the neutralizing antibody or polypeptide alone. Such inhibitors with the advantage of multiple biologics have the potential to be further developed as effective prophylactic and therapeutic agents, and may find wide application in treating viral diseases.

## Figures and Tables

**Table 1:** Binding kinetics analysis of fusion proteins to MERS-CoV S protein.

Fusion Proteins	*k*_on_ (M^−1^s^−1^)	*k*_off_ (s^−1^)	K_D_ (M)
CH3-pep	5.99 × 10^4^	6.56 × 10^4^	1.09
m336 scFv	4.66 × 10^5^	3.80 × 10^−4^	8.14 × 10^−10^
m336 scFv-pep	7.47 × 10^5^	1.20 × 10^−4^	1.60 × 10^−10^
m336 diabody-pep	1.14 × 10^6^	1.23 × 10^−3^	1.08 × 10^−9^
